# *Echinococcus granulosus sensu lato* promotes osteoclast differentiation through DUSP4-MAPK signaling in osseous echinococcosis

**DOI:** 10.3389/fmicb.2025.1558603

**Published:** 2025-03-19

**Authors:** Haohao Sun, Yaqing Liu, Yiping Huang, Kangjun Xiong, Zhendong Zhang, Weishan Wang, Yi Dai, Jing Li, Qi Li, Sibo Wang, Chenhui Shi

**Affiliations:** ^1^The First Affiliated Hospital of Shihezi University, Shihezi, China; ^2^The Medical College of Shihezi University, Shihezi, China; ^3^Xi’an Jiaotong University Affiliated Honghui Hospital, Xi’an, China

**Keywords:** *Echinococcus granulosus* (*E. granulosus*), bone, osteoclast, DUSP4, MAPK

## Abstract

**Introduction:**

Osseous echinococcosis, caused by *Echinococcus granulosus* infection, is characterized by progressive bone destruction driven by abnormal osteoclast activation. Dual-specificity phosphatase 4 (DUSP4), a key negative regulator of the MAPK pathway, inhibits osteoclast differentiation and bone resorption. This study aimed to elucidate the role of DUSP4 in *E. granulosus*-induced bone loss.

**Methods:**

In vitro, a co-culture system of *E. granulosus* protoscoleces (PSCs) and bone marrow-derived macrophages (BMMs) was established. Osteoclast differentiation and bone resorption were assessed using TRAP staining and F-actin immunofluorescence. Transcriptome sequencing identified DUSP4 as a key regulator. DUSP4 overexpression was performed to evaluate its effects on osteoclast markers and MAPK signaling (ERK, JNK, p38). In vivo, a mouse model of osseous echinococcosis was developed, and DUSP4 overexpression was achieved via lentiviral transduction. Bone destruction was analyzed using X-ray, micro-CT, and histology.

**Results:**

PSCs significantly enhanced osteoclast differentiation and bone resorption, upregulated osteoclast markers (CTSK, NFATc1), and activated MAPK signaling. DUSP4 overexpression reversed these effects, reducing osteoclast activity and MAPK phosphorylation. In vivo, PSC infection caused severe bone destruction, which was mitigated by DUSP4 overexpression.

**Disscussion:**

This study reveals the molecular mechanism by which *Echinococcus granulosus* drives abnormal osteoclast activation through the DUSP4-MAPK signaling axis. Parasitic infection suppresses DUSP4 expression, relieving its negative regulation of the MAPK pathway and leading to excessive osteoclast differentiation. Restoring DUSP4 expression effectively reverses abnormal MAPK pathway activation, reducing osteoclast bone resorption activity to physiological levels. These findings not only provide new insights into the pathological mechanisms of bone destruction in osseous echinococcosis but also establish DUSP4 as a critical therapeutic target for pathological bone resorption, laying the groundwork for host-directed treatment strategies for parasitic bone diseases.

## 1 Background

Cystic echinococcosis (CE) is a zoonotic disease caused by the larval stage of the fine-grained tapeworm *Echinococcus granulosus* (*E. granulosus*). Following accidental ingestion of *E. granulosus* eggs through contaminated food/water, the eggs hatch into oncospheres within the digestive tract. These larvae subsequently penetrate the intestinal wall, enter the bloodstream, and disseminate to various organs where they develop into metacestodes. Ultimately, these larval forms establish fluid-filled cysts containing infectious protoscoleces (PSC) (Kern et al., 2017). The typical structure of metacestodes is a nearly round cystic body, composed of a double-layered cyst wall and cyst contents: the outer keratinized layer serves a protective function, and the inner germinal layer; the cyst contents include daughter cysts, brood capsules, protoscoleces that grow out of the germinal layer and cyst fluid. It is worth noting that the host tissue forms a fibrous pericyst around the parasite (Carabajal et al., 2022). While it most commonly affects the liver, it can also involve other tissues and organs, including the lungs, lymph nodes, greater omentum, central nervous system, skin, retroperitoneum (including paravertebral region), and bone (Barth and Casulli, 2021).

Osseous echinococcosis, a severe manifestation of CE, is one of the most complex and devastating forms of the disease, with the cost of patient care and loss of livestock production placing a non-negligible economic burden on the global community (Budke et al., 2006; Alvarez et al., 2014). It is clinically characterized by an insidious onset and a single diagnostic and therapeutic tool, accompanied by a high rate of disability, recurrence and mortality (Monge-Maillo et al., 2017; Carvalho et al., 2021; Luan et al., 2023). Cattaneo et al. (2019) proposed the following three hypotheses: (1) mechanical compression by cysts leads to bone destruction; (2) cystic compression of blood vessels causes ischemic bone injury; (3) an increase in osteoclasts at the site of infection enhances bone resorption. Recent studies have identified osteoclast overactivation as a major cause of osteolytic changes in osseous echinococcosis (Sun et al., 2023). However, the molecular mechanisms underlying this pathology remain poorly understood, necessitating further research to identify therapeutic targets and elucidate disease pathways.

Osteoclast differentiation and function are mainly regulated by two cytokines, nuclear factor κB ligand receptor activator (RANKL) and macrophage colony-stimulating factor (M-CSF) (Ikeda and Takeshita, 2016). M-CSF drives the proliferation of osteoclast precursors, while the combination of M-CSF and RANKL induces their differentiation into tartrate-resistant acid phosphatase (TRAP)-positive multinucleated osteoclasts (Guo et al., 2020). RANKL binds to RANK on the surface of osteoclast precursors, forming the RANKL-RANK-TRAF6 complex, which activates signaling molecules and transcription factors, including NF-κB, mitogen-activated protein kinase (MAPK) (ERK, JNK, p38), and phosphoinositide 3 kinase (PI3K)/protein kinase B (Akt) (Nakashima et al., 2011). These pathways, in turn, activate transcription factors such as cytoplasmic nuclear factor of activated T-cells 1 (NFATc1) and activator protein 1 (AP-1), driving the expression of osteoclast effector molecules, such as cathepsin K(CTSK) and TRAP, which collectively mediate osteoclast differentiation and function. Notably, inhibiting RANKL signaling has been shown to impede osteoclastogenesis, offering potential therapeutic avenues for osteoclast-related diseases (Xiao et al., 2020; Li et al., 2022; Liu et al., 2023).

Transcriptome sequencing of PSC-treated bone marrow-derived mononuclear cells (BMMCs) revealed significant enrichment of genes involved in osteoclast differentiation, along with downregulation of dual-specificity phosphatase 4 (DUSP4) and activation of the MAPK signaling pathway. DUSP4, also known as mitogen-activated protein phosphatase 2 (MKP-2), dephosphorylates threonine (T) and tyrosine (Y) residues (T-X-Y motifs) on MAPK (ERK, JNK, P38) (Low and Zhang, 2016; Zhou et al., 2023; Li and Chen, 2021). The DUSP family is widely recognized for its role as a tumor suppressor in lung, breast, colorectal, and pancreatic cancers (Keyse, 2008; Aughton et al., 2022). Recent studies suggest that DUSP proteins regulate immune responses and bone metabolism. For example, IL-3 induces Stat5 activation to promote DUSP1 and DUSP2 expression, inhibiting osteoclast bone resorption (Hirose et al., 2014). Similarly, DUSP5 has been shown to inhibit LPS-induced osteoclast maturation and pro-inflammatory effects (Moon et al., 2014), while DUSP4 modulates the immune response in *Leishmania* protozoa infection (Al-Mutairi et al., 2010). However, whether DUSP4 directly affects osteoclast differentiation or bone loss has not yet been clarified.

In this study, we investigated the role of DUSP4 in osteoclast differentiation, its involvement in PSC-mediated effects, and its impact on bone loss *in vivo*. We observed that DUSP4 expression decreased during osteoclast differentiation, while its upregulation inhibited osteoclast activity and reduced bone loss. Mechanistically, DUSP4 inhibits MAPK phosphorylation, thereby attenuating osteoclast maturation and function. These findings suggest that DUSP4 may serve as a novel therapeutic target for osseous echinococcosis and related osteoclast-mediated bone diseases.

## 2 Materials and methods

### 2.1 Ethical approval and model construction

Male C57BL/6J mice (8 weeks old) were purchased from Wuhan Mouse Lai Bao Biotechnology Co., Ltd. (Wuhan, China) and housed at the Laboratory Animal Center of Shihezi University (Shihezi, China). All procedures involving animals were approved by the Animal Protection Committee of Shihezi University (Approval No. A2023-202-01). To construct the femoral osseous echinococcosis model, mice were anesthetized with sevoflurane (anesthesia concentration: 4–5%, maintenance concentration: 1–3%). The hairs on the skin surface from the lower end of their femur to the knee joints were shaved, and the operative area was sterilized using iodine povidone. 0.5 mL of PSC-containing RPMI-1640 complete medium (8,000 PSC/mL; Gibco) was drawn using a 1 mL syringe. The syringe was inserted through the knee joint and advanced approximately 1 cm retrogradely along the femoral subperiosteum toward the femoral head for injection. Control mice received an equal volume of saline under identical conditions.

### 2.2 Extraction and culture of *Echinococcus granulosus* protoscoleces

Sheep livers naturally infected with *E. granulosus* were purchased from slaughterhouses around Shihezi City, China. In the laboratory, the livers were cleaned of surface blood stains and sterilized with 75% alcohol, and cystic fluid was aspirated from the vesicles using a 50 mL syringe under aseptic conditions. Only clear and translucent cystic fluid was retained. The fluid was fully precipitated, the supernatant removed, and then repeatedly washed and re-precipitated using sterile PBS, and transferred to culture flasks and cultured with RPMI-1640 complete medium at 37°C, with half medium changes every 2 days.

### 2.3 Culture and differentiation of BMMCs into osteoclasts

Bone marrow cells were isolated from the femurs and tibias of C57BL/6 mice by flushing the bone marrow cavity with *α*-MEM essential medium (Gibco; Thermo Fisher Scientific, Inc.) supplemented with 10% fetal bovine serum (FBS) (Gibco; Thermo Fisher Scientific, Inc.). The cells were incubated at 37°C with 5% CO₂ overnight. Suspended cells were collected as BMMCs and further cultured in the presence of M-CSF (R&D Systems, Inc.) and RANKL (R&D Systems, Inc.), and the medium was changed every 2 days until mature osteoclasts were formed.

### 2.4 Co-culture of PSC and osteoclast

BMMCs were plated and allowed to adhere. Cells were then treated with PSCs at four graded concentrations (0, 50, 100, 200 particles/mL) in α-MEM complete medium supplemented with 10% FBS, 30 ng/mL M-CSF, and 100 ng/mL RANKL. RNA was extracted after 24 h of culture, followed by protein extraction at 72 h. Mature osteoclasts, characterized by multinucleated and fused morphology, were typically observed under microscopy after approximately 5 days. Cells were subsequently washed to remove PSCs for staining procedures.

### 2.5 RNA-seq

After 24-h co-culture of PSCs with BMMCs, total RNA was extracted using TRIzol reagent (control group: BMMCs without PSCs). Flash-frozen samples were transported to MetWare (Wuhan, China) for sequencing analysis. Following RNA integrity assessment using Qsep 400 Bioanalyzer, strand-specific libraries were constructed with Illumina TruSeq Stranded Total RNA Library Prep Kit (20020598) using 200 ng RNA as template. Gene expression quantification was performed using DESeq2 v1.38.3, with differentially expressed genes (DEGs) identified at thresholds of |log2FC| >1 and FDR <0.05.

### 2.6 TRAP staining

Adherent osteoclasts were washed twice with PBS and fixed with 4% paraformaldehyde for 30 min. After another PBS wash, cells were stained using the TRAP-Kit 387A (Sigma-Aldrich, Merck KGaA) following the manufacturer’s instructions. TRAP-positive mature osteoclasts were identified as purplish-red cells with more than 3 nuclei under a light microscope.

### 2.7 F-actin staining

Adherent osteoclasts were washed twice with PBS and fixed with 4% paraformaldehyde for 30 min. After another PBS wash, cells were permeabilized with 0.1% Triton X-100. Cells were stained with the F-actin kit (Abcam, #ab112125) according to the manufacturer’s instructions. F-actins were visualized as green rings under a fluorescence microscope.

### 2.8 Lentiviral infection of BMMCs

Lentiviral overexpression constructs for DUSP4 (LV-DUSP4) and control (LV-NC) were obtained from Gemma (Shanghai, China). BMMCs were transfected with LV-DUSP4 and LV-NC lentiviruses in the presence of M-CSF (30 ng/mL). Transduction efficiency was assessed using fluorescence microscopy after 24 h, and overexpression efficiency was evaluated via real-time quantitative PCR (RT-qPCR) and western blot.

### 2.9 Cell viability and proliferation assay

Cell viability was measured using the Cell Counting Kit-8 (CCK-8; MCE, United States) to compare the proliferative potential of BMMCs. BMMCs infected with lentivirus were seeded in 96-well plates at a density of 5,000 cells per well. The absorbance of each well was measured at 450 nm (OD450). The mean of three replicates recorded each day was used to construct growth curves.

### 2.10 Western blot

BMMCs were lysed with cell lysis buffer containing 1% protease and phosphatase inhibitors (Roche, Germany). Proteins were separated by 10%SDS polyacrylamide gel electrophoresis and transferred to a PVDF membrane (Merck Millipore, Germany). The membrane was blocked with 5% bovine serum albumin (BSA) blocking solution (BIOTEK, China) for 2 h, and then incubated overnight at 4°C with primary antibodies: DUSP4 (1:1000; Boster, #OT17C11); GAPDH (1:2000; Boster, #BG-7) ERK (1:2,000; #4695), p-ERK (1:1,000; #4370), JNK (1:2,000; #9252), p-JNK (1:1,000; #4668), p38 (1:2,000; #8690), p-p38 (1:1,000; #4511) (Cell Signaling Technology, Inc.); MMP-9 (1:1000; #ab76003); c-Fos (1:2,000; #ab190289), TRAP (1:1,000; #ab191406), NFATc1 (4 μg/mL; #ab2796), Cathepsin K (1:2,000; #ab19027) (Abcam, Inc). The next day, membranes were incubated with secondary antibodies: AffiniPure Goat Anti-Mouse IgG (1:20,000; Boster, BA1038), AffiniPure Mouse Anti-Rabbit IgG (1:20,000; Boster, BA2020) for 2 h at room temperature. Finally, we incubated the membranes with ECL solution (1:1 mixture, BioSharp, BL523B) and captured images using a chemiluminescence imaging system (Tanon-4600, Tanon, China).

### 2.11 Real-time quantitative PCR

Total RNA was extracted from primary cells cultured in osteoclast induction medium for 24 h using an RNA extraction kit (#RC112-01, Vazyme Biotech, China). Complementary DNA (cDNA) was synthesized using a reverse transcription kit (#11141ES60, YEASEN). RT-qPCR amplification was then performed using the following specific primers:

**Table 1:** 

	Forward	Reverse
MMP9	F′ GGACCCGAAGCGGACATTG	R′ CGTCGTCGAAATGGGCATCT
TRAP	5′-TACCTGTGTGGACATGACC-3′	5′-CAGATCCATAGTGAAACCGC-3′
NFATc1	5′-GGTCTTCCGAGTTCACATCC-3′	5′-CACAGGTCCCGGTCAGTC-3′
CTSK	5′-TGGACTATACCCAGGGAAACCTC-3′	5′-CAAGTAACTATGATGCCCAAGCAG-3′
DUSP4	5′-CGTGCGCTGCAATACCATC-3′	5′-CTCATAGCCACCTTTAAGCAGG-3′
DUSP2	5′-TGCTGGGGCCGAAAATAGC-3′	5′-CATAGATCGGAACTCACCTGGT-3′
β-actin	5′-GTCGTACCACAGGCATTGTGATGG-3′	5′-GCAATGCCTGGGTACATGGTGG-3′

### 2.12 Cellular immunofluorescence

BMMCs were seeded in 96-well plates at a density of 1 × 10^5^ cells per well to induce osteoclast differentiation. Cells were fixed with 4% formaldehyde for 30 min, permeabilized with PBS containing 0.1% Triton X-100, and washed with PBS. Subsequently, cells were blocked with 5% BSA for 30 min at room temperature and incubated overnight at 4°C with an anti-DUSP4 primary antibody. The following day, cells were incubated with goat anti-rabbit IgG H&L antibody (Alexa Fluor^®^ 488, Abcam; 1:100, ab150077) for 2 h at room temperature. F-actin staining was performed by incubating the cells with the appropriate reagent for 30 min, followed by DAPI staining (Sigma Aldrich; Merck KGaA, GER) for 5 min. Finally, cells were washed three times with PBS and observed using an inverted fluorescence microscope (Olympus Corporation, Japan).

### 2.13 Micro-CT analysis

After 6 months of model establishment, the mice were euthanized under anesthesia (inject 40% carbon dioxide into the transparent execution chamber, expose the mice for 5 min and then observe for another 2 min to confirm their death), and their femurs were bilaterally collected and fixed with 4% paraformaldehyde for preservation. Histomorphometric analysis of the mouse femurs was performed using a microcomputed tomography (μ-CT) system (Scanco Medical) in a uniform manner. Scanning was performed at 100 kV and 98 mA, with a resolution of 10 μm per pixel. Finally, bone parameters were analyzed and 3D reconstructed using the built-in software of μ-CT.

### 2.14 Histological staining

Tissue sections were dewaxed, rehydrated, and subjected to hematoxylin and eosin (H&E) staining for general histological observation. TRAP staining was performed by immersion staining using TRAP working solution. After staining, the sections were sealed with neutral gum and then examined under a microscope for imaging.

### 2.15 Data analysis

All data were statistically analyzed and visualized using GraphPad Prism 9.0 software. Each experiment was performed with at least three independent replicates. Data were expressed as mean ± standard deviation (±SD). Comparisons between two groups were performed using independent samples t-test, and comparisons between multiple groups were performed using one-way or two-way analysis of variance (ANOVA). Statistical significance levels are indicated by ^*^p < 0.05, ^**^p < 0.01, ^***^p < 0.001, and ^****^p < 0.0001, respectively.

## 3 Results

### 3.1 PSC promotes RANKL-dependent osteoclast differentiation

**Figure 1:**
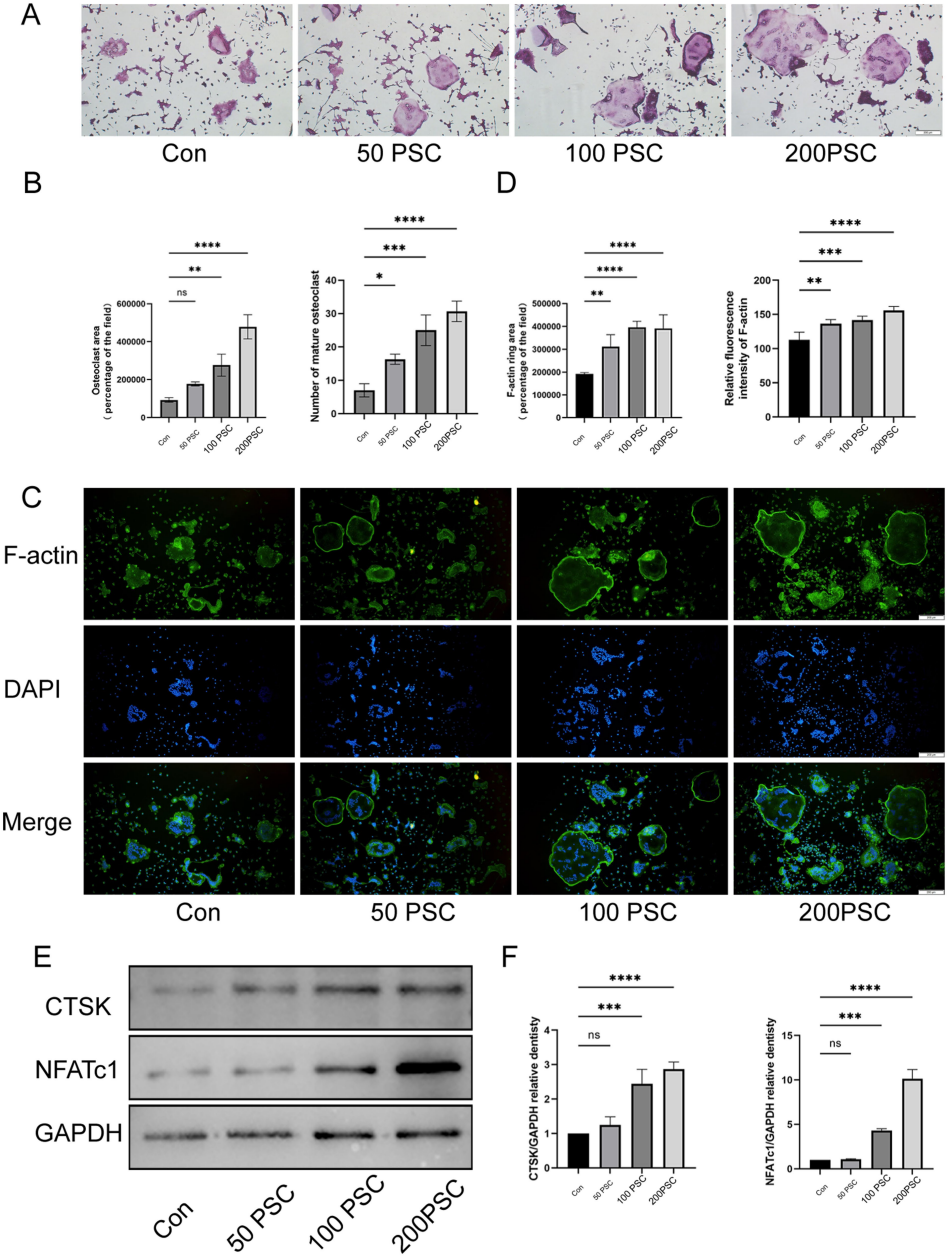
PSC promotes the maturation and differentiation of BMMCs into osteoclasts. **(A)** TRAP staining of BMMCs co-cultured with different concentration of PSC. Mature osteoclasts were identified as purple-red cells with ≥3 nuclei. Magnification ×40. **(B)** Quantification of the number and area of mature osteoclasts shown in **(A)**. **(C)** F-actin staining of BMMCs after 5 days of co-culture with different concentration of PSCs. Green: F-actin; blue: DAPI. Magnification ×40. **(D)** Quantification of the area and fluorescence intensity of the F-actin ring shown in **(C)**. **(E)** Protein expression of CTSK and NFATc1 in BMMCs after 36 h of intervention with different concentration of PSCs. **(F)** Quantification of the protein bands of CTSK and NFATc1 in **(E)**. Data are representative of three independent experiments. ns, not significant; ^*^p < 0.05, ^**^p < 0.01, ^***^p < 0.001, and ^****^p < 0.0001.

TRAP staining revealed that co-culture of PSC with osteoclasts significantly increased the number and area of mature osteoclasts compared to the control group, in a concentration-dependent manner (Figures 1A,B). Mature osteoclasts were surrounded by a well-defined F-actin ring, a structure essential for bone resorption. Immunofluorescence staining further demonstrated that PSC intervention significantly promoted the formation of multinucleated osteoclasts (Figure 1C). In the high-dose PSC group, the F-actin ring displayed a typical large ring structure, whereas the RANKL group exhibited significantly smaller F-actin rings and fewer nuclei within the rings (Figures 1C,D). Western blot analysis demonstrated that PSC intervention elevated the expression of cathepsin K (CTSK), an osteoclast-related functional protein, in a concentration-dependent manner. Additionally, the protein expression of NFATc1, a transcription factor that is also related to osteoclast differentiation, was also significantly upregulated (Figures 1E,F).

### 3.2 Transcriptomic analysis reveals PSC impacts DUSP and MAPK Signaling pathways

**Figure 2:**
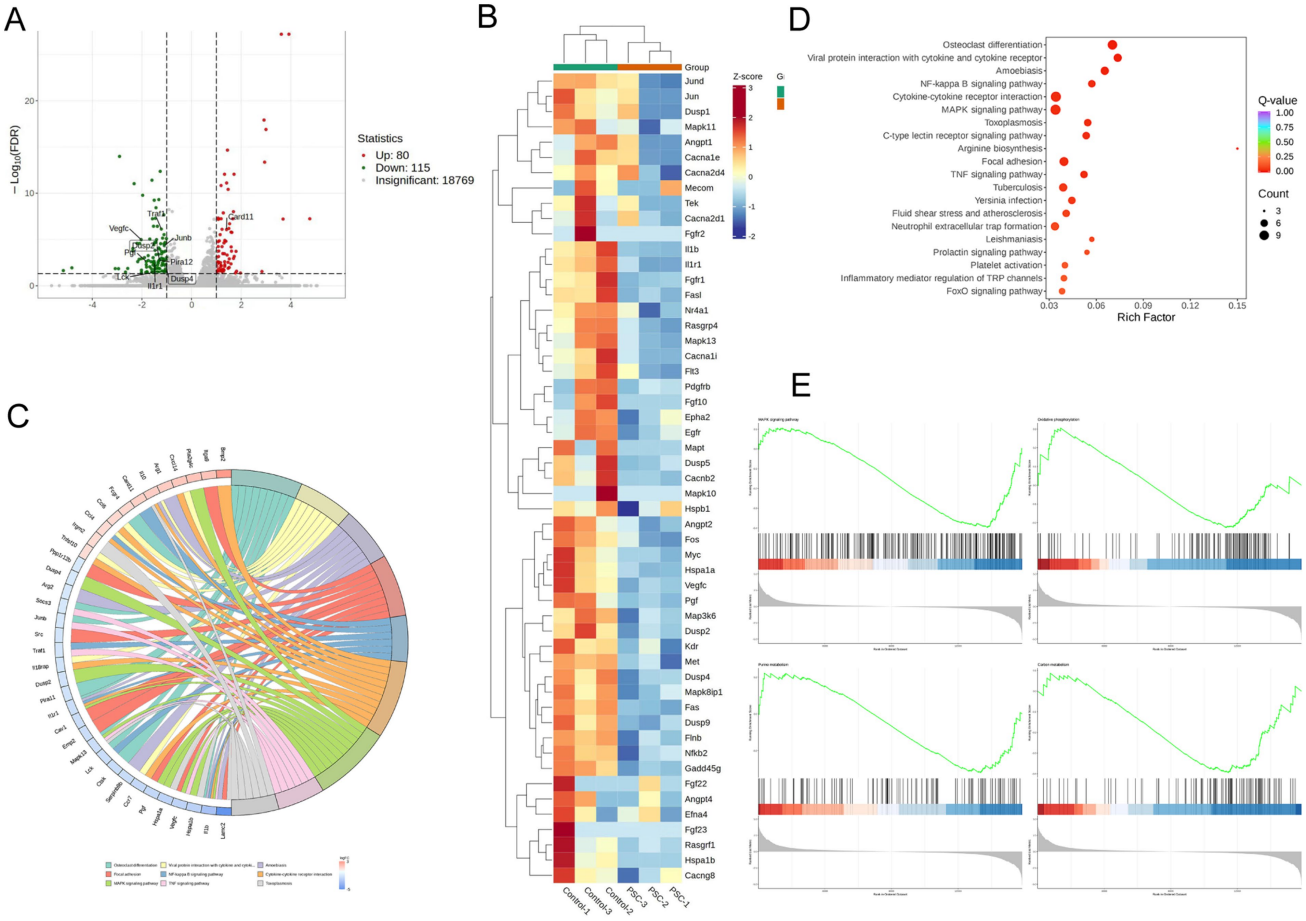
PSC intervention affects osteoclast-associated pathways. **(A)** Volcano plot showing differentially expressed genes (DEGs) between PSC-intervened and normally cultured BMMCs. **(B)** KEGG pathway analysis of DEGs. **(C)** Top 10 altered signaling pathways and associated DEGs. **(D)** Gene clustering analysis of DEGs. **(E)** GSEA analysis comparing PSC-intervened BMMCs to normally cultured BMMCs.

To investigate the potential molecular mechanism of PSC-mediated regulation of osteoclast maturation and differentiation, we performed transcriptome sequencing analysis on RANKL-treated BMMCs cells under normal induction or co-cultured with PSC. Using log2FC| ≥1 and *p* < 0.05 as the criteria for screening differentially expressed genes (DEGs), 195 DEGs were found to be up-/down-regulated in PSC intervention group (Figure 2A). We performed KEGG analysis with adjusted *p* < 0.05 as a screening criterion and identified the top 10 enriched biological processes and related pathways. These included osteoclast differentiation, cytokine-cytokine receptor interaction, MAPK signaling, cell adhesion, cAMP signaling, NF-κB signaling, TNF signaling, Rap1 signaling, FoxO signaling, and neutrophil extracellular TRAP formation (Figures 2B,C). Under normal induction with RANKL, the MAPK and NF-κB pathways were identified as the primary pathways driving osteoclast differentiation. KEGG analysis revealed significant enrichment of the MAPK signaling pathway among DEGs between the two groups, in which DUSP2 and DUSP4 of the DUSP family were downregulated (Figures 2C,D). GSEA analysis (Figure 2E) confirmed significant changes in MAPK signaling pathway and oxidative phosphorylation, highlighting their involvement in PSC-mediated osteoclast differentiation.

### 3.3 PSC intervention induces DUSP4 downregulation and MAPK activation

**Figure 3:**
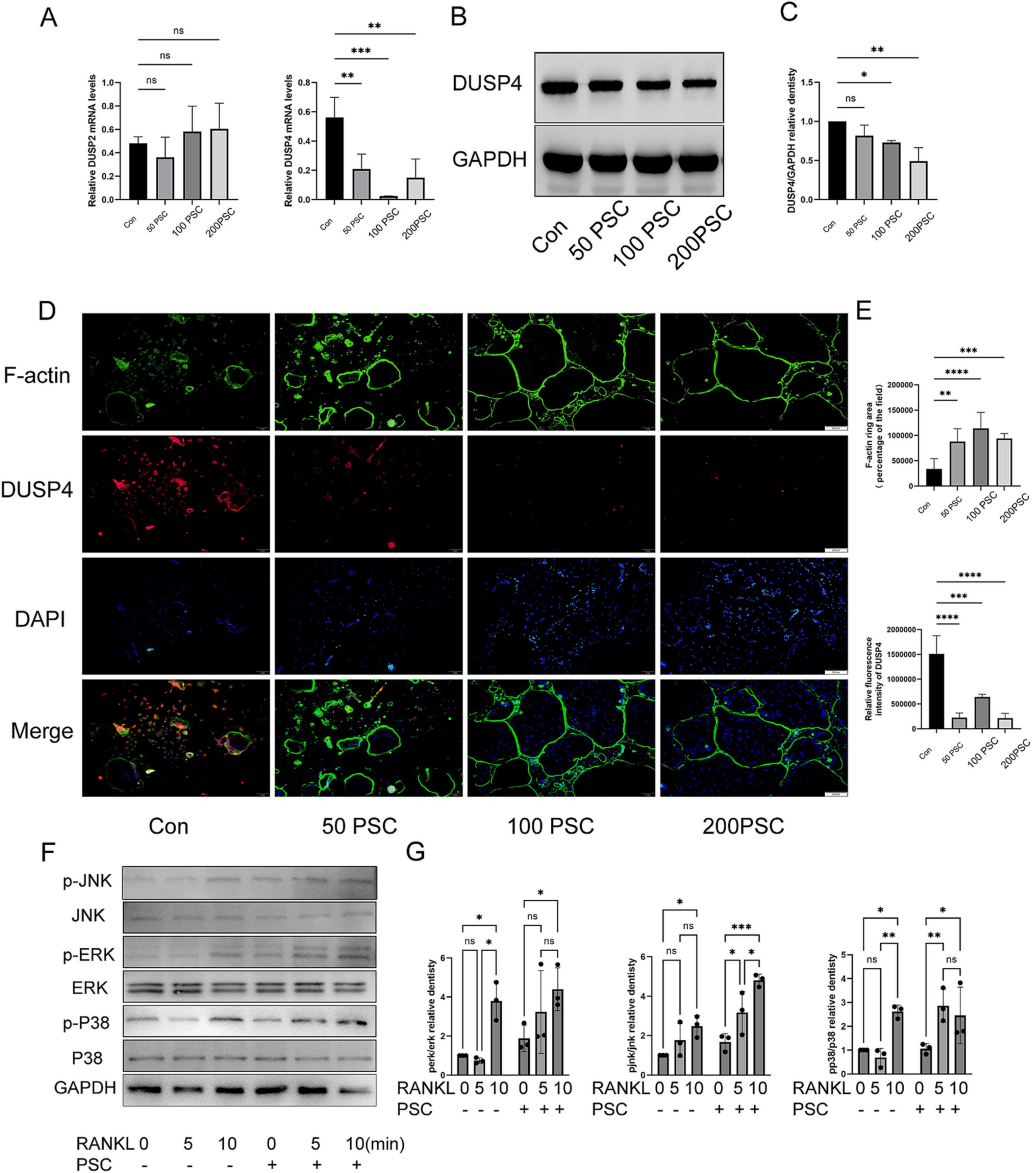
PSC intervention downregulates DUSP4 and upregulates the MAPK signaling pathway. **(A)** mRNA expression levels of DUSP2 and DUSP4 following different doses of PSC intervention. **(B)** Protein expression levels of DUSP4 after different doses of PSC intervention. **(C)** Quantification of DUSP4 protein levels shown in **(B)**. **(D)** Visualization of F-actin ring formation and DUSP4 expression in osteoclasts after different doses of PSC intervention. Green: F-actin; red: DUSP4; blue: DAPI. Magnification ×40. **(E)** Quantification of the F-actin ring area and the absolute and relative fluorescence intensity of DUSP4 shown in **(D)**. Data represent three independent experiments. ns, not significant; ^*^p < 0.05, ^**^p < 0.01, ^***^p < 0.001, and ^****^p < 0.0001.

Expression of DUSP2 and DUSP4 was analyzed during osteoclast differentiation. RT-qPCR analysis revealed that DUSP2 expression remained unchanged after PSC intervention, whereas DUSP4 expression decreased in a dose-dependent manner with increasing PSC concentrations (Figure 3A), suggesting that the downregulation of DUSP4 may lead to activation of BMMCs. Subsequent western blot analysis confirmed the reduction in DUSP4 expression following PSC intervention (Figures 3B,C). Immunofluorescence staining further demonstrated that with RANKL induction, PSC treatment not only enhanced osteoclast formation but also decreased DUSP4 expression levels (Figures 3D,E). Additionally, PSC intervention significantly increased the phosphorylation of ERK, JNK and p38 at different time points (Figures 3F,G). These results suggest that PSC promotes MAPK signaling activation.

### 3.4 Overexpression of DUSP4 inhibits PSC-induced osteoclast differentiation

**Figure 4:**
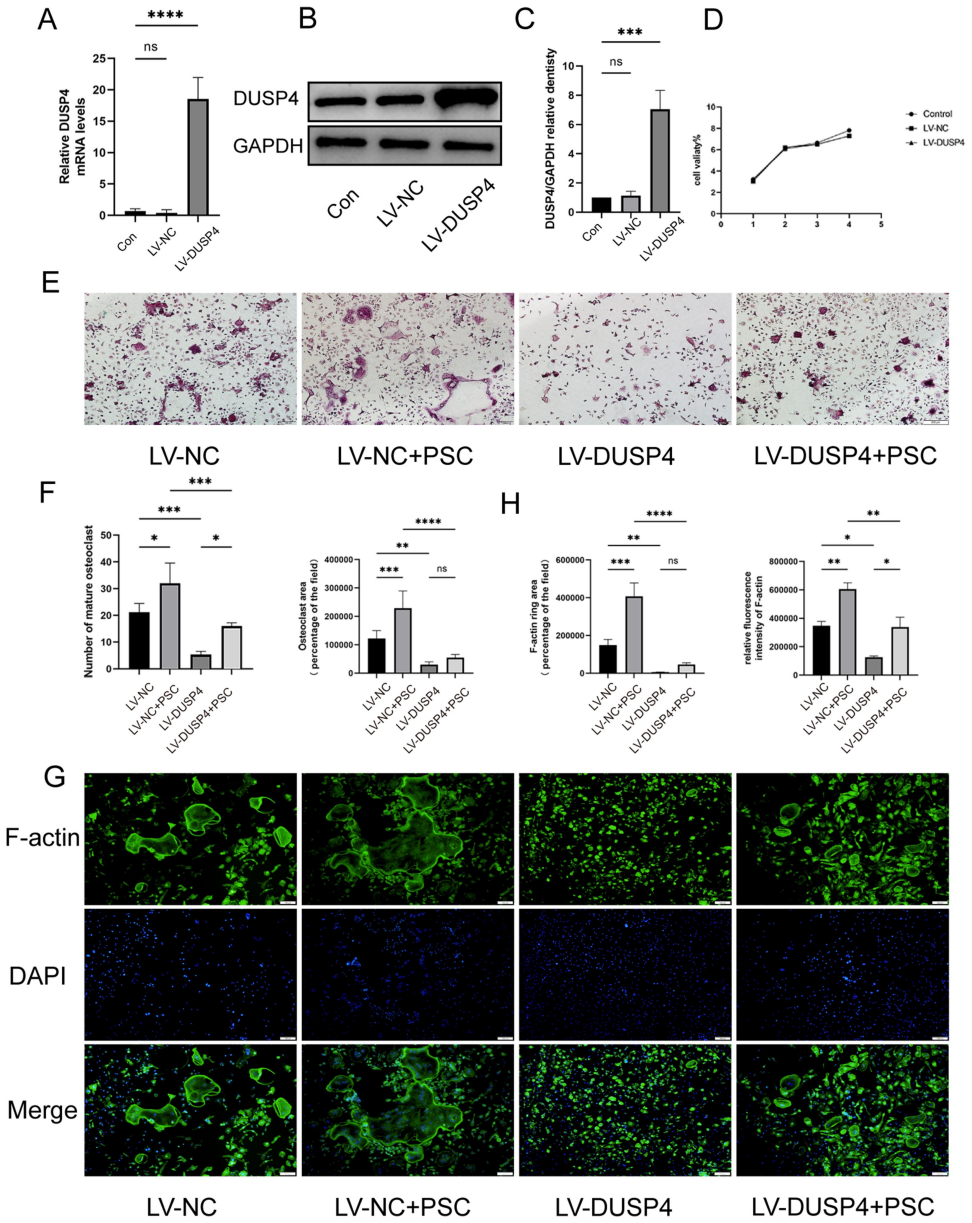
Overexpression of DUSP4 affects osteoclast formation. **(A)** mRNA expression levels of DUSP4 in control, lentiviral-transfected null, and DUSP4-overexpression groups. **(B)** Protein levels of DUSP4 in the same groups. **(C)** Quantification of **(B)**. **(D)** Cell viability assay in the same groups. **(E)** TRAP staining of osteoclasts in control and DUSP4-overexpression groups after PSC intervention. Magnification ×40. **(F)** Quantification of the area occupied by TRAP-positive cells and the number of TRAP-positive cells shown in **(E)**. **(G)** F-actin ring staining in control and DUSP4-overexpression groups after PSC intervention green: F-actin; blue: DAPI. Magnification ×40. **(H)** Quantification of the F-actin ring area and fluorescence intensity shown in **(G)**. Data represent three independent experiments, and significance was determined using one-way ANOVA. ns, no significance; ^*^p < 0.05, ^**^p < 0.01, and ^***^p < 0.001.

BMMCs were transfected with lentiviral vectors LV-NC (negative control) or LV-DUSP4 (to overexpress DUSP4), followed by culture with RANKL (50 ng/mL) and M-CSF (30 ng/mL). Untreated controls (no transfection, with cytokines) were included. DUSP4 overexpression was demonstrated by RT-qPCR and western blot (Figures 4A–C). CCK-8 assays showed no significant differences in viability between LV-NC, LV-DUSP4, and controls (Figure 4D). In the LV-NC group, the number and area of TRAP-positive osteoclasts increased significantly after the addition of PSC, while the number of osteoclasts in the LV-DUSP4 group decreased, but partially recovered after the addition of PSC (Figures 4E,F). This trend is consistent with the results of F-actin staining area (Figures 4G,H), indicating a critical role of DUSP4 in inhibiting osteoclast differentiation and suggesting that PSC may antagonize the inhibitory effect of DUSP 4 on osteoclast differentiation.

### 3.5 DUSP4 overexpression attenuates PSC-induced MAPK activation and osteoclast differentiation

**Figure 5:**
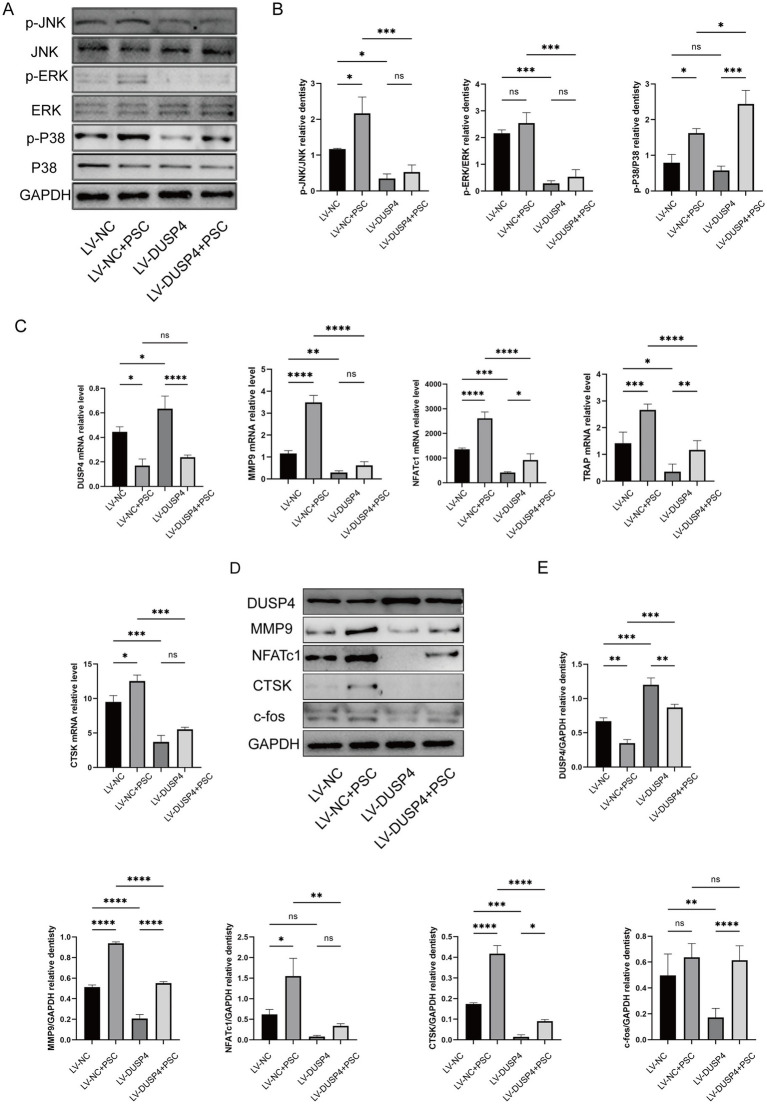
Effects of DUSP4 on the MAPK pathway and osteoclast-related genes. **(A)** Western blot analysis showing changes in MAPK signaling pathway components (p-JNK, p-ERK, and p-p38) in osteoclasts after PSC intervention in control and DUSP4-overexpression groups. **(B)** Quantification of MAPK pathway phosphorylation levels in osteoclasts from control and DUSP4-overexpression groups, as shown in **(A)**. **(C)** mRNA expression levels of osteoclast-related proteins (DUSP4, MMP9, NFATc1, TRAP, and CTSK) after PSC intervention in control and DUSP4-overexpression groups. **(D)** Western blot analysis of osteoclast-related proteins (DUSP4, MMP9, NFATc1, CTSK, and c-fos) after PSC intervention in control and overexpression groups. **(E)** Quantification of osteoclast-related proteins expression levels in **(D)**. Data represent three independent experiments, and significance was determined using one-way ANOVA. ns, no significance;^*^p < 0.05, ^**^p < 0.01, and ^***^p < 0.001.

The null, DUSP4-overexpression, and control groups described above were treated with PSC to evaluate the impact on MAPK activation and osteoclast differentiation. Phosphorylation levels of p38 (p-p38) and JNK (p-JNK) were elevated after PSC intervention compared to the control group, while phosphorylation level of ERK (p-ERK) remained unchanged. However, in the DUSP4-overexpression group, the phosphorylation levels of ERK, JNK and p38 were reduced compared to the null group (Figures 5A,B). The study further examined the effects of PSC and DUSP4 on the differentiation and function of RANKL-induced osteoclasts. RT-qPCR and western blot analyses revealed that expression levels of NFATc1, CTSK, MMP9, and other osteoclast-related proteins aligned well with changes observed in phosphorylation level of MAPK signaling pathway components (Figures 5C–E). These findings suggest that DUSP4 modulates osteoclast differentiation and function by attenuating PSC-induced MAPK signaling.

### 3.6 DUSP4 overexpression mitigates bone destruction in osseous echinococcosis *in vivo*

**Figure 6:**
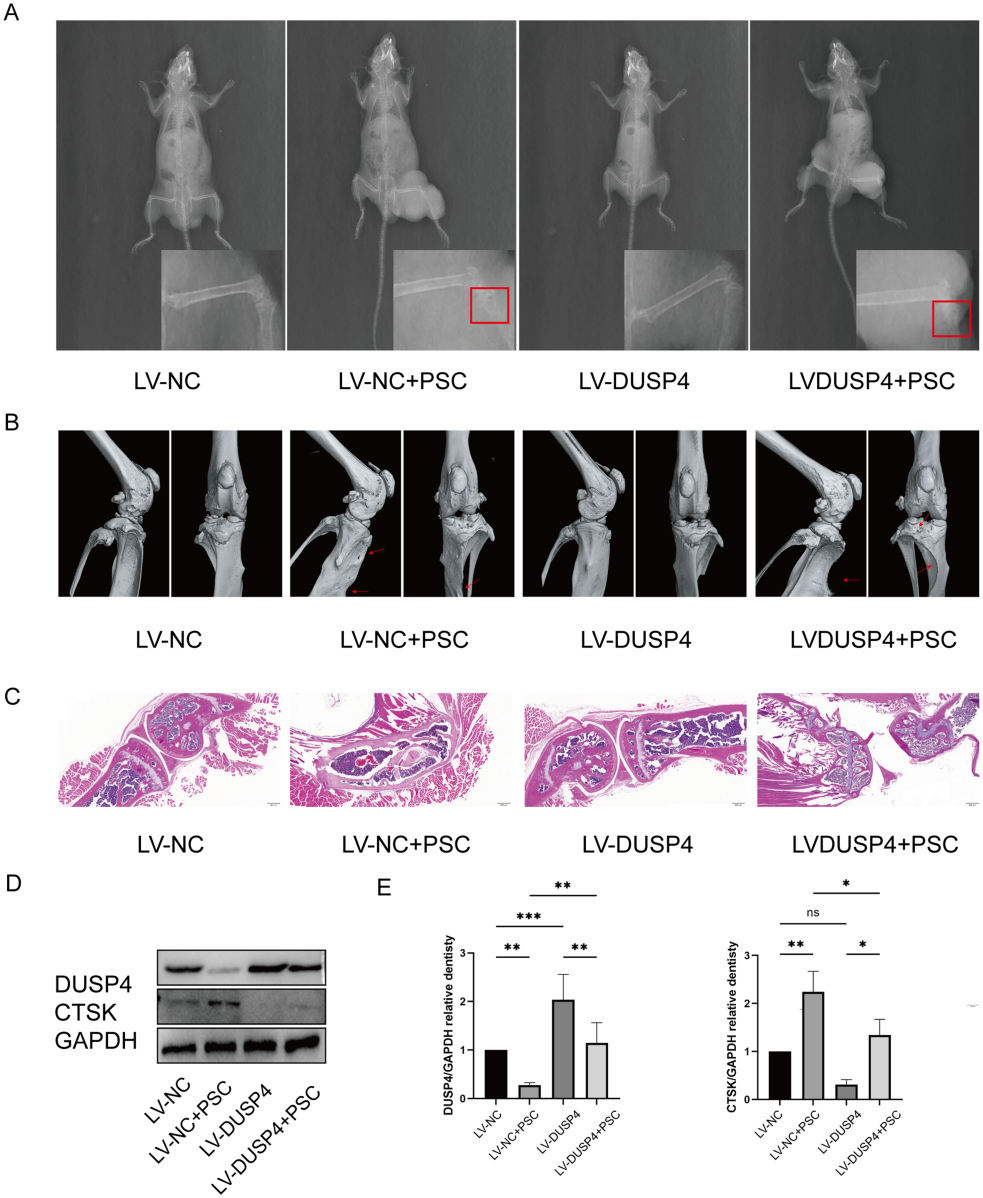
DUSP4 inhibits osteoclasts and attenuates bone destruction in osseous echinococcosis. **(A)** X-ray examination of long bones from mice treated with empty vector, lentiviral DUSP4, or left untreated in the affection of PSC after 6 months, with red rectangle indicating cyst. **(B)** Micro-CT images of long bones from each treatment group, with red arrows indicating bone defects. **(C)** HE staining of bone tissue sections from each treatment group. Magnification ×20. **(D)** Western blot analysis of osteoclast-related proteins (DUSP4 and CTSK) after PSC intervention in control and DUSP4-overexpression groups. **(E)** Quantification of osteoclast-related protein expression levels in osteoclasts from control and DUSP4-overexpression groups, as shown in **(D)**. Data are presented as mean ± SD from five mice per group. ^*^p < 0.05, ^**^p < 0.01, and ^***^p < 0.001.

To determine whether DUSP4 inhibits osteolytic lesions, mice undergoing PSC intervention for 4 months were treated with lentivirus expressing DUSP4 (LV-DUSP4) or empty lentivirus (LV-NC). Following euthanasia, tibial specimens were subjected to micro-CT and histological analyses. X-ray imaging revealed no significant changes in the size of the encapsulated vesicles after lentiviral treatment. However, damage to the bone cortex was evident in the modeling group, indicating that the mouse model of bone encapsulosis was successfully constructed (Figure 6A). Micro-CT scans showed that mice treated with LV-DUSP4 exhibited reduced bone destruction compared to those treated with LV-NC (Figure 6B). HE staining revealed disrupted trabecular bone structure in Echinococcus-infected mice, while LV-DUSP4 treatment partially rescued this structural damage (Figure 6C). Western blot analysis of femoral protein extracts demonstrated that Echinococcus infection suppressed DUSP4 expression, which was successfully restored by LV-DUSP4 overexpression (Figures 6D,E). Additionally, the osteoclast marker protein CTSK was upregulated following infection but returned to baseline levels after DUSP4 overexpression, indicating a protective effect against infection-induced bone resorption (Figures 6D,E).

## 4 Discussion

CE is a globally distributed zoonotic disease, with bone involvement occurs in approximately 4% CE cases, and its diagnosis and therapeutic management are challenging (Deplazes et al., 2017; Monge-Maillo and Lopez-Velez, 2023). This form of the disease is characterized by fragile bones and an increased risk of fractures, leading to high rates of disability and mortality, as well as substantial economic losses (Neumayr et al., 2013). Current treatment options for osseous echinococcosis are limited. Medications such as albendazole and mebendazole are commonly used; however, their long-term use is not recommended due to side effects, including muscle and joint pain and osteonecrosis of the jaw. Surgical interventions are considered when drug therapy is ineffective or when complete removal of cysts is not feasible, but these procedures are not suitable for long-term treatment due to high recurrence rate. Therefore, there is an urgent need for alternative and complementary long-term therapies with fewer side effects for the treatment of clinical osseous echinococcosis diseases. Developing early diagnostic biomarkers and identifying therapeutic targets are essential steps in this process. Recent studies have highlighted the pivotal role of osteoclasts in the pathogenesis of osseous echinococcosis (Sun et al., 2023; Huang et al., 2025; Wang, 2024). Overactivation of osteoclasts has been identified as a critical factor in disease progression, providing valuable insights into its underlying mechanisms (Sun et al., 2023). On this basis, our study revealed that the DUSP4 and MAPK signaling pathways may serve as potential therapeutic targets for osseous echinococcosis, offering new avenues for treatment strategies.

Osteoclasts are multinucleated giant cells originating from the monocyte/macrophage lineage of the hematopoietic system. They play an indispensable role in bone remodeling and maintaining skeletal homeostasis through their unique bone resorption function (Soe et al., 2021; Veis and O’Brien, 2023). Early studies using labeled nucleotides showed that osteoclasts have a lifespan of only days to weeks in the body (Jaworski et al., 1981). Due to the short lifespan of osteoclasts, any change that extends their lifespan and enhances their viability may lead to increased osteoclast activity and, consequently, enhanced bone resorption activity (Li et al., 2023). In this study, we found that PSC could promote the formation of RANKL-induced osteoclasts in a concentration-dependent manner.

The functional states of osteoclasts are diverse and dynamically regulated to adapt to changes in the microenvironment (Florencio-Silva et al., 2015). Osteoclast signaling molecules (e.g., kinases and phosphatases) play important roles in bone homeostasis and diseases such as osteoporosis by inducing cytokines or chemokines (Kim et al., 2020). Our transcriptome sequencing analysis revealed that osteoclasts subjected to PSC intervention were significantly enriched in pathways related to osteoclast differentiation. Among these, mitogen-activated protein kinases (MAPKs) were identified as crucial signaling components. The MAPK signaling pathway, comprising p38 (p38α, p38β, p38γ/δ), JNK (JNK1, JNK2, JNK3), ERK (ERK1, ERK2), and ERK5, is involved in the pathogenesis of bone homeostasis imbalance, and regulates various cellular activities, including gene expression, mitosis, differentiation, cell survival, apoptosis, inflammation, stress, and immune responses (Sun et al., 2021). RANKL treatment of BMMCs induces phosphorylation of ERK, p38, and JNK. And treatment of the same cells with PSC enhances these phosphorylation events, providing strong evidence that PSC mediates its effects on osteoclast differentiation and function via activation of the ERK, p38, and JNK signaling pathways.

MAPK inhibitors have been developed as potential treatments to suppress osteoclast activity; however, none have progressed to phase III trials due to limited efficacy or adverse side effects (Poulikakos and Solit, 2011; Chuang and Tan, 2019). Studies of these MAPK kinase inhibitors suggest that upstream signaling molecules may be more effective therapeutic targets than downstream signaling molecules. Thus, upstream regulators of MAPK may serve as promising biomarkers or therapeutic targets for osseous echinococcosis. MAPK phosphatases (MKPs), including serine–threonine phosphatases (PP2A and PP2C), tyrosine phosphatases (PTPN5, PTPN7, and PTPRR), and dual-specificity phosphatase (DUSP) family members, play critical roles in modulating MAPK activity (Lang et al., 2006). Transcriptome sequencing in this study revealed significant downregulation of DUSP2 and DUSP4 in the MAPK signaling pathway, with DUSP4 showing pronounced downregulation in osteoclasts with PSC intervention, as confirmed by RT-qPCR. In this study, we explored the potential application of DUSP4 in osteoclasts as a biomarker and/or therapeutic target for osseous echinococcosis. DUSP4 (also known as MKP2) is a key MAPK regulator that inactivates JNK, p38, and ERK (Mandal et al., 2023). Overexpression of DUSP4 also inhibits the phosphorylation of STAT5, while DUSP4 deficiency results in enhanced STAT5 phosphorylation/activation in T cells (Scotto et al., 2020). MAPKs are widely regarded as important therapeutic targets for inflammatory diseases, including osteoporosis (Ullah et al., 2022; Gao et al., 2023), but the involvement of DUSP4 in osteoclast MAPK regulation has not been previously reported. In this study, we demonstrated that DUSP4 expression in osteoclasts was significantly decreased at the mRNA and protein levels following PSC intervention. Immunofluorescence analysis further confirmed attenuation of DUSP4 signaling during PSC-induced osteoclastogenesis. Notably, DUSP4 overexpression suppressed RANKL-induced osteoclast formation and significantly inhibited the activation of ERK, JNK, and p38. In addition, several inflammatory cytokines, including IL-3, IL-4, IL-6, and TNF, are known to negatively regulate osteoclastogenesis by inactivating JNK and p38 (Amarasekara et al., 2018). This suggests that DUSP4 and inflammatory cytokines may play complementary roles in modulating JNK and p38 activity, thereby affecting osteoclast function.

Osseous echinococcosis is a parasitic disease that that spreads through the bloodstream, affecting multiple organs and tissues. Osteoclasts, which are derived from hematopoietic stem cells, exhibit dysregulated signaling pathways in patients with *E. granulosus* infection (Liu et al., 2022). Aberrant, downregulation or overexpression of these signaling molecules could serve as valuable diagnostic biomarkers for osseous echinococcosis. DUSP family proteins, known for their regulatory roles in various disease contexts, such as chronic infections, autoimmune diseases, cancers, and age-related disorders, may hold promise as a druggable target over existing therapies that primarily work by manipulating protein kinase activity (An et al., 2021). Previous studies have reported increased macrophages, dendritic cells (DCs), regulatory T cells (Tregs), and myeloid-derived suppressor cells (MDSCs) in *Echinococcus granulosus-*infected BALB/c mice (Cao et al., 2020; Zhang et al., 2022). Like other helminths, *E. granulosus* induces host immune responses through parasite-derived antigenic proteins, exosomes, and miRNAs that shift macrophage polarization from a pro-inflammatory M1 state to an anti-inflammatory M2 phenotype (Zhang et al., 2022; Wang et al., 2023). This polarization plays a critical role in osteoclast formation (Kushioka et al., 2023). In this study, we verified *in vivo* the promotional effect of *E. granulosus* on bone resorption through a mouse model of osteoclastic disease. Importantly, overexpression of DUSP4 effectively attenuated bone destruction caused by osteoclasts in the infected mice. These findings highlight the potential of DUSP4 as a promising therapeutic target for inhibiting osteoclast activity and mitigating bone destruction *in vivo*.

Our experimental findings specifically support the Cattaneo’s third hypothesis, demonstrating that *E. granulosus* infection promotes osteoclast differentiation through the DUSP4-MAPK signaling axis. However, as appropriately noted, this mechanism does not preclude contributions from mechanical or ischemic factors, particularly in cases involving non-fertile cysts where compressive effects may dominate. While our current model focuses on osteoclast-driven resorption, we acknowledge that a comprehensive understanding of bone destruction in CE requires systematic investigation.

## Data Availability

The original contributions presented in the study are publicly available. This data can be found here: https://www.ncbi.nlm.nih.gov/sra/PRJNA1226371.
